# First Report of Focal Atrial Tachycardia Originating From the Left Atrial Appendage in a 13‐Year‐Old Girl Treated Using the OPTRELL Multipolar Mapping Catheter

**DOI:** 10.1002/joa3.70153

**Published:** 2025-07-22

**Authors:** Ryo Nakagawa, Hiroshi Furusho, Toshihiko Yasuda, Raita Araki

**Affiliations:** ^1^ Department of Pediatrics Ishikawa Prefectural Central Hospital Ishikawa Japan; ^2^ Department of Cardiology Ishikawa Prefectural Central Hospital Ishikawa Japan

**Keywords:** catheter ablation, focal atrial tachycardia, left atrial appendage, multipolar mapping, pediatrics

## Abstract

Safe and effective mapping of focal atrial tachycardia from the left atrial appendage using an array catheter enabled successful ablation in a pediatric patient without complications.

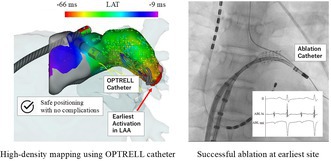

Focal atrial tachycardia (AT) predominantly originates from the crista terminalis and tricuspid annulus in the right atrium. Focal AT originating from the left atrial appendage (LAA) is relatively rare and carries a high risk of tachycardia‐induced cardiomyopathy (TIC) [1]. The thin walls of the LAA also present a high risk of perforation during catheter ablation. In focal arrhythmias, multipolar mapping can be used to analyze a large number of electrograms in a short period compared with conventional point‐by‐point mapping and is safe and effective [2]. The array catheter (OPTRELL; Biosense Webster, Diamond Bar, USA) with 36 or 48 electrodes allows for bipolar recordings in two orthogonal planes to identify the impulse propagation directions. Ventricular arrhythmia and atrial fibrillation mapping have been performed using the OPTRELL and high‐density (HD) grid catheters, respectively, which share design; however, their use in mapping focal AT originating from the LAA remains to be investigated. We present a case wherein catheter ablation using an array catheter was successfully and safely performed for a focal AT originating from the LAA.

A 13‐year‐old female, weighing 46 kg, with no significant medical history was referred after frequent premature atrial contractions were detected during routine school cardiac screening. She had also experienced mild chest discomfort for the past 1 to 2 years, but did not consider it necessary to visit a hospital. The 12‐lead electrocardiogram (ECG) revealed incessant AT (Figure 1) with a tachycardia cycle length (TCL) of 632 ms. The polarity of the P waves in AT was negative in lead I, bifid in lead II, positive in lead V1, and positive in the inferior leads, suggesting that the origin was the left pulmonary veins or the LAA, based on the P wave algorithm [3]. Transthoracic echocardiography revealed no functional impairment or structural abnormalities. NT‐proBNP was measured at 24 pg/mL. The Holter ECG monitoring indicated AT throughout the day. After discussing pharmacological therapy and catheter ablation, the patient and her family opted for catheter ablation. Preoperative contrast‐enhanced computed tomography revealed a normal morphology of the LAA.

**FIGURE 1:**
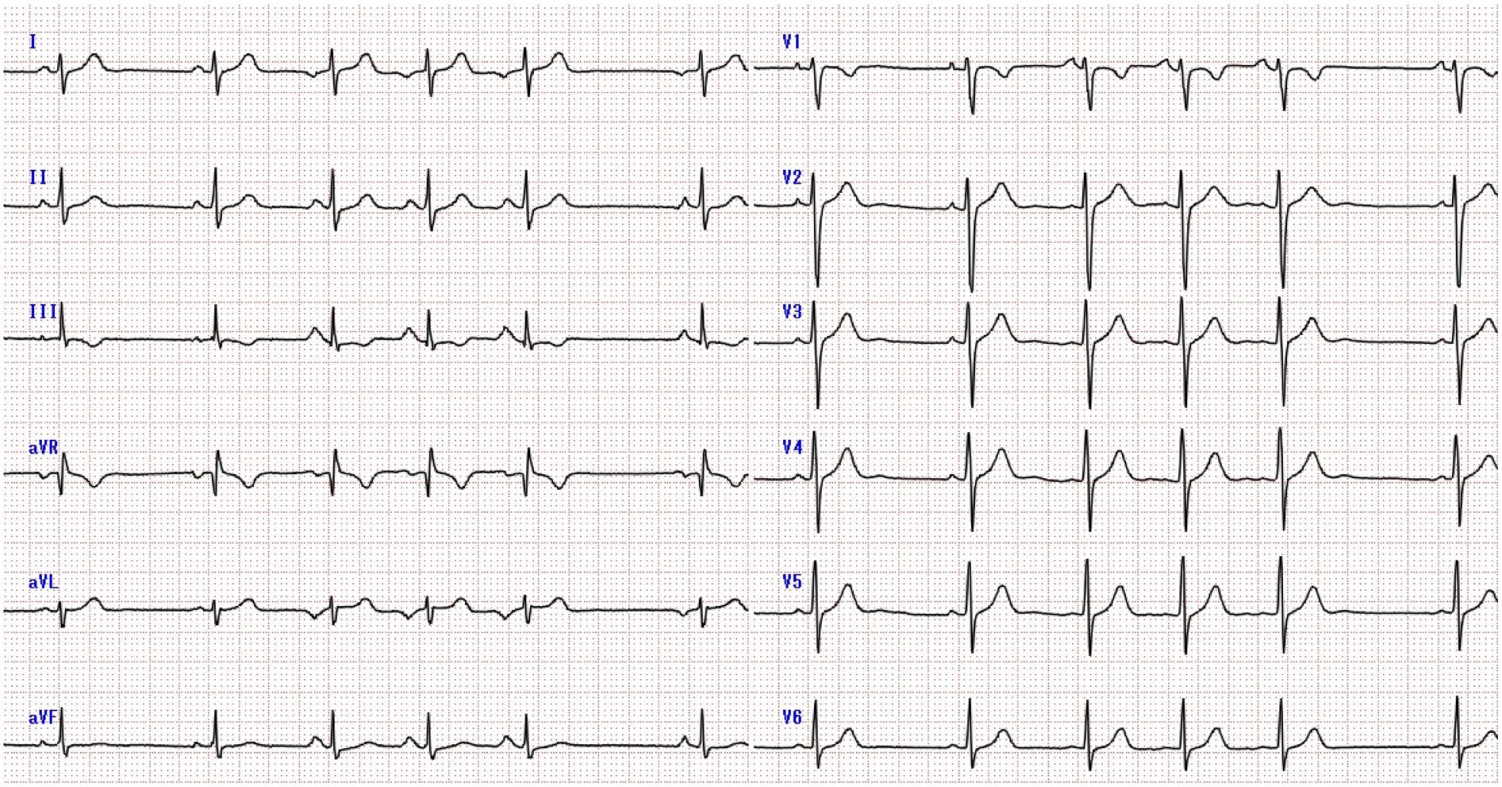
Twelve‐lead electrocardiogram of the patient. Atrial tachycardia is of the incessant type, with a tachycardia cycle length of 632 ms. The polarity of the P wave in atrial tachycardia is negative in lead I, bifid in lead II, positive in lead V1, and positive in the inferior leads. The setting was 5.00 mm/mV, 25.0 mm/s.

During catheter ablation, the patient was sedated with dexmedetomidine. Although sedation reduced the frequency of AT, it was still inducible with isoproterenol stimulation. The earliest coronary sinus (CS) activation was observed in the distal CS. Transseptal puncture was performed using an RF needle (NRG^TK^ Transseptal Needle; Boston Scientific, Natick, MA, USA). LAA angiography was performed before mapping the LAA (Figure 2a). To differentiate the origin between the LAA and the left upper pulmonary vein (LUPV), we carefully assessed the spatial distribution of the earliest activation and propagation sequence. Activation mapping was performed using an OPTRELL in the three‐dimensional mapping system (CARTO3; Biosense Webster) with a long sheath (STOUT; Medikit Co. Ltd., Tokyo, Japan). It showed earlier activation in the LAA than the LUPV. The earliest electrogram was recorded at the distal portion of the LAA. Activation mapping in the LAA was completed in 6 min and revealed a centrifugal pattern from the distal part of the LAA, leading to a diagnosis of focal AT originating from the LAA (Figure 2b). A total of 2158 mapping points (i.e., electrograms recorded by the OPTRELL catheter) were collected during the activation mapping.

**FIGURE 2:**
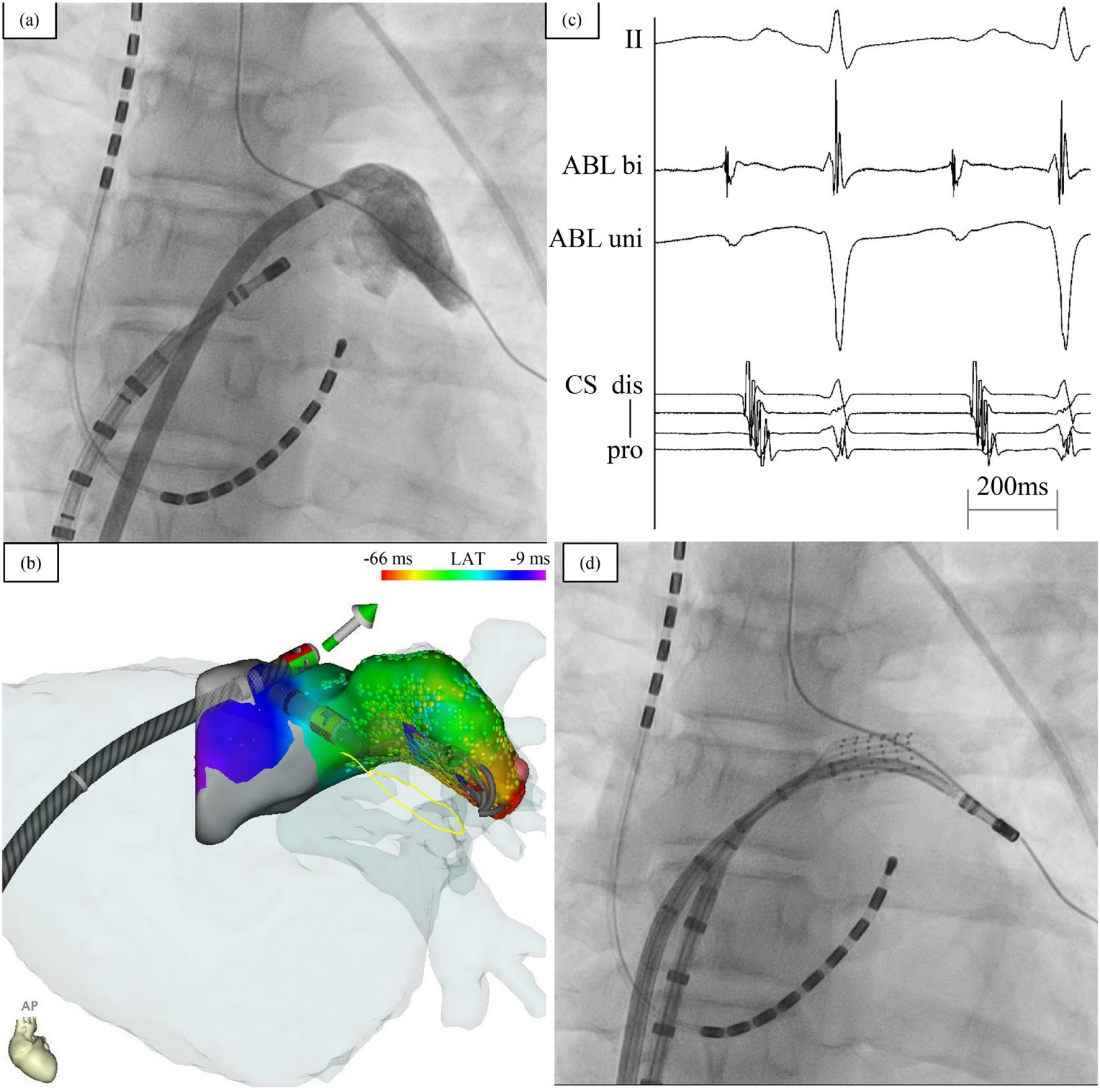
(a) Fluoroscopic image of the left atrial appendage angiography. (b) High‐density activation map of the left atrial appendage showing the position of OPTRELL mapping catheter. (c) Lead II surface electrocardiogram and intracardiac electrograms recorded from the ablation and coronary sinus (CS) catheters. The earliest CS activation was observed in the distal CS. The intracardiac electrogram of the ablation catheter at the earliest activation site preceded the P wave in lead II by 68 ms. (d) Catheter position during ablation within the left atrial appendage. CS, coronary sinus; LAA, left atrial appendage.

Subsequently, mapping of the earliest site was performed using an open‐irrigated contact force‐sensing catheter (Navistar ThermoCool SmartTouch; Biosense Webster) with a steerable sheath (VIZIGO; Biosense Webster). The earliest atrial activation preceded the P wave by 68 ms (Figure 2c). At the identified earliest site, repetitive bumps were observed, and AT could no longer be induced by isoproterenol or programmed stimulation. Thus, four points of ablation were performed at the earliest site and its surrounding area during sinus rhythm (Figure 2d). Ablation was performed at a power of 25 W, with an average temperature of 29°C, a total radiofrequency application of 293 s, and an irrigation flow rate of 8 mL/min. The contact force during ablation ranged from 2 to 61 g (mean 13 g). Impedance monitoring showed a decrease from 136 to 128 Ω during energy delivery. Thereafter, AT could no longer be induced with isoproterenol or programmed stimulation. Subsequently, the procedure was concluded. Postprocedure intracardiac echocardiography indicated no signs of pericardial tamponade, and the ablation site was confirmed to be in the distal part of the LAA. Transthoracic echocardiography after the procedure also revealed no deterioration in cardiac function and no findings suggestive of pericardial tamponade. Holter monitoring performed 3 months after catheter ablation revealed a slight recurrence of atrial tachyarrhythmia. Supraventricular arrhythmia accounted for only 1.0% of the total heart beats, and the longest episode of atrial tachycardia consisted of 219 consecutive beats. As the patient remained asymptomatic, outpatient follow‐up was planned.

Focal AT is a relatively rare arrhythmia. According to the German Ablation Registry, only 3.4% of all ablation cases had focal AT [4]. In pediatric patients, the frequency of LAA origin is similarly low, around 3% [5]. In a previous report of 13 cases of AT originating from the LAA, all foci were located at the base of the appendage, and no distal origin was documented. Therefore, our case may represent a less common distal LAA origin [6]. Because of the complex anatomy and thin walls of the LAA, catheter manipulation is challenging with an increased risk of cardiac perforation. In cases where catheter ablation is not feasible or has failed, surgical resection of the LAA may be considered as an alternative treatment [7]. Approximately 10% of patients with focal AT may develop TIC. In a study by Medi et al. summarizing TIC in focal AT, risk factors for TIC are incessant type, younger age, male sex, long TCL, and slow ventricular rates, and they reported that 84% of LAA‐originating focal AT cases were of the incessant type, and 42% showed signs of heart failure [1]. This case involved a young patient whose AT was of the incessant type, originated from the LAA, had a long TCL, and was associated with a slow ventricular rate, suggesting a high risk of TIC. In this case, the patient did not seek medical attention despite the symptoms and only visited the hospital after a school heart screening. If not for the school heart screening, it is highly likely that the patient would have developed TIC. Although TIC did not ultimately develop in this patient, this case highlights the utility of the OPTRELL high‐density mapping catheter in accurately identifying the earliest activation site within the LAA and the safety of radiofrequency catheter ablation using a contact force‐sensing catheter, even in the anatomically complex structure of the LAA.

During ablation treatment for focal AT, it is crucial to accurately assess the earliest activation site. Recently, HD mapping has been increasingly used. Chieng et al. reported that in focal arrhythmias, the use of the HD grid catheter reduces the treatment and mapping time compared with point‐by‐point mapping. However, no significant difference was observed in the acute success rates or mid‐term outcomes, and they suggested that randomized trials are needed to determine whether multipolar mapping improves the prognosis of focal arrhythmias [8].

We safely performed mapping using the array catheter and successfully conducted ablation therapy for focal AT originating from the LAA. However, further research is warranted to confirm the effectiveness of the multipolar mapping catheter in focal AT.

## Ethics Statement

The authors have nothing to report.

## Consent

Written informed consent was obtained from the patient for publication of this case report and accompanying images.

## Conflicts of Interest

The authors declare no conflicts of interest.

## Data Availability

The authors have nothing to report.
